# Genome-Wide Identification of N^6^-Methyladenosine (m^6^A) SNPs Associated With Rheumatoid Arthritis

**DOI:** 10.3389/fgene.2018.00299

**Published:** 2018-08-03

**Authors:** Xing-Bo Mo, Yong-Hong Zhang, Shu-Feng Lei

**Affiliations:** ^1^Center for Genetic Epidemiology and Genomics, School of Public Health, Medical College of Soochow University, Suzhou, China; ^2^Jiangsu Key Laboratory of Preventive and Translational Medicine for Geriatric Diseases, Soochow University, Suzhou, China; ^3^Department of Epidemiology, School of Public Health, Medical College of Soochow University, Suzhou, China

**Keywords:** rheumatoid arthritis, M^6^A, genome-wide association study, major histocompatibility complex, gene expression

## Introduction

Previous large scale genome-wide association studies (GWAS) have identified more than one hundred susceptibility loci for rheumatoid arthritis (RA) (Stahl et al., 2010; Eyre et al., 2012; Okada et al., 2012, 2014b). One major issue in the post GWAS era is to identify functional (causal) variants in the disease-associated loci. Recently, whole-exome sequencing studies have identified several missense mutations for RA (Mitsunaga et al., 2013; Okada et al., 2014a). Nevertheless, few studies have focused on potentially functional variants which could affect N^6^-methyladenosine (m^6^A) methylation.

N^6^-methyladenosine (m^6^A) has been discovered as the first example of reversible RNA methylation and is a pervasive modification in the mRNA of all eukaryotes. It plays critical roles in regulating many fundamental biological processes such as gene expression control (Meyer and Jaffrey, 2014) and regulation of mRNA stability (Wang et al., 2014) and homeostasis (Edupuganti et al., 2017). Evidence has increasingly shown that m^6^A modification modulates almost all aspects of RNA processing such as nuclear export, translatability, splicing, and miRNA processing (Visvanathan and Somasundaram, 2018). Thus, m^6^A modification adds a new layer of gene expression regulation, and it has been implicated in T cell differentiation, homeostasis, and response to HIV infection (Li et al., 2018). Moreover, it was found to be involved in the etiology of various diseases (Visvanathan and Somasundaram, 2018). Given the vital function of m^6^A modification in gene expression regulation and immune response, it is possible that m^6^A is involved in the etiology of RA.

Recent studies have suggested that SNPs would affect m^6^A by altering the RNA sequences of the target sites or key flanking nucleotides (Zheng et al., 2018). It means that m^6^A-associated SNPs (m^6^A-SNPs) may have regulatory potential to affect gene expression and mRNA stability and homeostasis, which may consequently affect disease such as RA. However, what is the relationship between m^6^A-SNPs and RA is still unclear. Thus, we examined the effect of the m^6^A-SNPs on RA in a large scale GWAS. Then, to test whether the RA-associated m^6^A-SNPs have potential regulatory effects on gene expression and whether these disruptions may contribute to RA, we performed eQTL and differential expression analyses using whole genome data of about 10.5 million SNPs and 21,323 mRNAs from 26 RA cases and 17 controls, as well as public data.

## Value of the data

- We found 37 m^6^A-SNPs were associated with RA at genome-wide significance level (*P* < 5.0 × 10^−8^) in Asians or Europeans.
- A heap of RA-associated m^6^A-SNPs at the 6p21-6p22 which contains genes encode the major histocompatibility complex were detected, such as rs1033500 (*P* = 1.0 × 10^−250^) in *C6orf10* and rs1042136 (*P* = 2.5 × 10^−34^) in *HLA-DPB1*.
- We detected associations of 27 m^6^A-SNPs with expressions of 24 local genes. By integrating the RA-associated m^6^A-SNPs with gene expression data, we formed 23 SNP-Gene-RA trios.
- The present data increase our understanding on the regulation patterns of SNP, and may provide new insight into the mechanism underlying the associations between SNPs and RA.

## Materials and methods

### Determination of m^6^A-SNPs for RA

We first investigated the effect of m^6^A-SNPs on RA in data of a large scale GWAS (Okada et al., 2014b), which was publicly available at http://plaza.umin.ac.jp/~yokada/datasource/software.htm. This GWAS comprised 19,234 RA cases and 61,565 controls from European and Asian populations. The downloaded datasets contained summary results of almost 6.6 million SNPs. The m^6^A-SNPs in these 6.6 million SNPs were sorted out according to a list of m^6^A-SNPs in the m6AVar database (http://m6avar.renlab.org/). The m6AVar database currently contains 303,001 m^6^A-SNPs for human (Zheng et al., 2018). We have not validated the associations between the identified m^6^A-SNPs and RA in another independent sample. However, these SNPs were located in RA loci that were confirmed in previous GWAS, so the associations should be reliable.

### Study sample

Twenty-six female patients suffering from RA were included into this study. All patients had been diagnosed as RA following the 2010 American College of Rheumatology/European League Against Rheumatism (ACR/EULAR) criteria (Aletaha et al., 2010). In this study, the EULAR Disease Activity Score (DAS28) (Prevoo et al., 1995) of the 26 patients ranges from 2.91 to 6.41. The mean age of the patients was 47.39 (±10.71). A total of 17 female subjects without RA or other autoimmune diseases were recruited as controls. The mean age of the controls was 47.11 (±14.09). Genomic DNA and total RNA in PBMCs were extracted according to the instructions recommended by the manufacturer. The study was approved by the ethical committee of Soochow University. The written informed consents were obtained from all the subjects.

### Genotyping and mRNA expression profiling

Affymetrix Genome-Wide Human SNP Array 6.0 chips were employed for SNP genotyping by following the protocol recommended by the manufacturer. After quality control and genotype imputation by the 1000 Genomes project phase 3 reference panel using FISH software (Zhang et al., 2014), about 10.5 million SNPs could be used in the following analyses. Genome-wide mRNA expression was profiled using mRNA Human Gene Expression Microarray V4.0 (CapitalBio Corp, Beijing, China) according to the manufacturer's instructions. After further filtering out probes with detection rate less than 80% and/or incomplete annotation information, a total of 21,323 unique mRNAs were used for further analysis.

### eQTL analysis for the RA-associated m^6^A-SNPs

The RA-associated m^6^A-SNPs may have regulatory potential to affect gene expression and mRNA stability and homeostasis, which may result in variations of mRNA levels. We carried out the *cis*-acting eQTL analysis in PBMCs of the 43 subjects to obtain functional evidence for the identified m^6^A-SNPs. The m^6^A-SNPs affect m^6^A by altering the mRNA sequences of the target sites or key flanking nucleotides, so we only focused on the relationship of the m^6^A-SNP with the expression of the local gene. The distance from SNP position to either starting point of or ending point of the mRNA transcript was 0 bp (the tested m^6^A-SNPs all locate inside genes) in the *cis*-eQTL analysis, which was carried out by using the R package MatrixeQTL (Shabalin, 2012). We applied linear regression analyses to assess the associations between SNPs and mRNA expressions, adjusted for RA. In addition, we also performed the *cis*-eQTL analysis in the HaploReg database (Ward and Kellis, 2012). Except for eQTL effects, we searched for potential functionalities in transcription regulation, such as altering protein binding, in HaploReg and RegulomeDB for the identified m^6^A-SNPs. ENCODE data were also searched in UCSC Genome Browser to identify more information for the significant m^6^A-SNPs.

### Differential expression analysis

We further tried to determine if the disruption of gene expression by the RA-associated m^6^A-SNPs could affect RA. We examined the differential expression of the identified genes that the m^6^A-SNPs showed significant *cis*-eQTL signals with in our in-house dataset (26 RA cases and 17 controls) in PBMCs. Since the PBMCs consist of multiple immune cells including monocytes, CD8 T cells, B cells, regulatory T cells (Tregs), and activated natural killer (NK) cells, we further inferred the expressions of these 24 genes in various cell subgroups from PBMCs by using the CIBERSORT algorithm. The CIBERSORT algorithm is a method using support vector regression for cell type deconvolution. We did deconvolution for the 26 RA cases and 17 controls with 100 iterations and a default input matrix of cell-type specific gene expression signatures of 22 distinct immune cell types (LM22) (Newman et al., 2015).

Besides, we also detected differential expression genes based on three public gene expression datasets, GSE15573 (peripheral blood mononuclear cells) (Teixeira et al., 2009), GSE17755 (peripheral blood cells) (Lee et al., 2011), and GSE1919 (synovial tissues) (Ungethuem et al., 2010), which were downloaded from GEO database. Differential expression was tested by comparing mean gene expression signals between cases and controls using *t*-test. The significance level of *P* = 0.05 was used for the differential expression analyses. Differential expression genes detected in at least one of the three studies were considered.

## Results and discussion

### RA-associated m^6^A-SNPs

**Figure 1:**
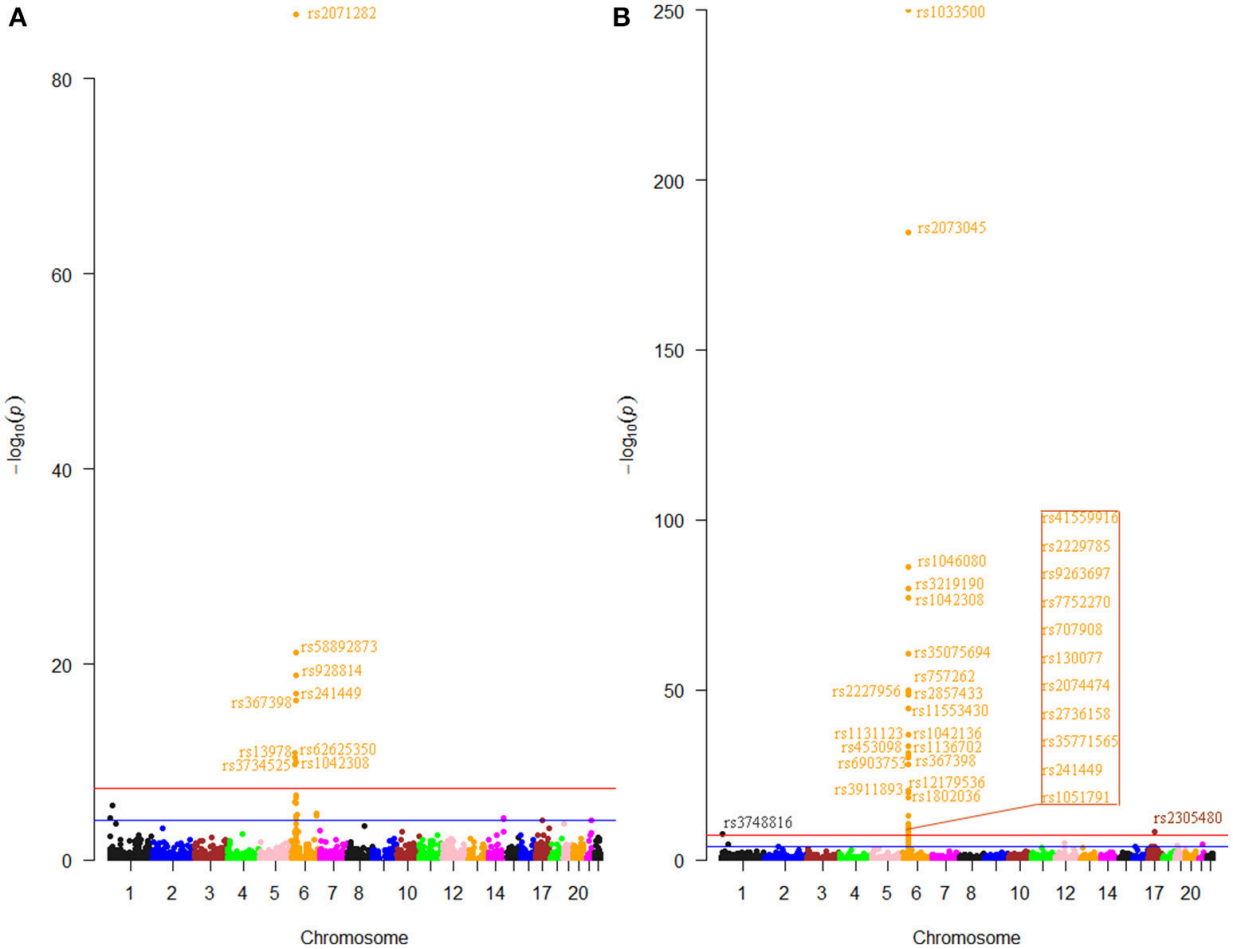
Genome-wide results for the association between m^6^A-SNPs and RA. The Manhattan plots show –log_10_*P* values for the association of 2,672 m^6^A-SNPs with RA in Asians **(A)** and the association of 3,667 m^6^A-SNPs with RA in Europeans **(B)**. The data was from the RA GWAS published at 2014. SNPs reached the genome-wide significance level (*P* < 5.0 × 10^−8^) were annotated.

The first step of this study was to pick out m^6^A-SNPs from the RA GWAS dataset which containing 6.6 million SNPs according to the annotation information of the 313 thousand m^6^A-SNPs in the m6AVar database. We found 3,883 unique m^6^A-SNPs which located not only in protein-coding genes, but also non-coding RNAs. Among these SNPs, 227 and 308 (476 unique) were nominally (*P* < 0.05) associated with RA in Asians and Europeans, respectively. Specifically, 9 and 32 (38 unique) m^6^A-SNPs were significant at genome-wide level (*P* < 5.0 × 10^−8^) in Asians (Figure 1A) and Europeans (Figure 1B), respectively. Among these genome-wide significant m^6^A-SNPs, the effects of rs1046080, rs13978, rs2736158, and rs58892873 on m^6^A were confirmed by miCLIP/PA-m^6^A-Seq experiments. The effects of another 8 SNPs on m^6^A were confirmed by MeRIP-Seq experiments. The rest 26 fell within the low confidence categories (predicted to be associated with m^6^A). The effects of the predicted SNPs on m^6^A modification have not been validated by technical and biological experiments. Further experiments are needed to determine their effects in large scale studies (e.g., QTL mapping).

Most of these SNPs were in the major histocompatibility complex (MHC) region, except rs3748816 (*P* = 2.8 × 10^−8^, *MMEL1*) and rs2305480 (*P* = 5.0 × 10^−9^, *GSDMB*) (Supplementary Table S1). The m^6^A-SNP rs3748816 in *MMEL1* at chromosome 1 was very near in physical with the reported SNP chr1:2523811. Similarly, the m^6^A-SNP rs2305480 in *GSDMB* at chromosome 17 located next to the reported SNP chr17:38031857. Although studies have made efforts to define the association across this region and identify functional and potentially causal variants, pinpointing those loci were still challenging, in part due to the complexity and the broad linkage disequilibrium characteristic of the MHC (Raychaudhuri et al., 2012). Our study illustrated how identification of m^6^A-SNPs from the GWAS dataset can help fine-map association signals in the MHC region.

In addition, we examined the associations of SNPs in the key m^6^A regulators encoded genes *METTL3, METTL14, WTAP, FTO*, and *ALKBH5* with RA. Unfortunately, very few SNPs in these genes were examined in the original GWAS and we found no genome-wide significant association. The only one SNP with *P* < 0.0001 was rs55982358 in *FTO* (*P* = 5.6 × 10^−5^ in Europeans). Five SNPs in *METTL3*, 1 SNPs in *METTL14*, 1 SNPs in *WTAP*, 159 SNPs in *FTO* and 21 SNPs in *ALKBH5* with *P* < 0.05 were found for Asian or European population. These SNPs were not in the list of m^6^A-SNPs in the m6VAR database, but they should also be m^6^A associated because genetic variants in these genes might have potentials to influence the key m^6^A proteins.

### eQTL analysis

To further clarify the possible functional mechanisms underlying the identified m^6^A-SNPs in association with RA, we investigated whether they were associated with the expression of local genes (Supplementary Tables S4 and S5). In total, 27 (13 for Asians and 20 for Europeans) of the examined RA-associated m^6^A-SNPs (*P* < 0.0001 in Asians or Europeans) showed *cis*-eQTL (SNP affects gene expression at the same locus) signals with the 24 corresponding genes in different cells or tissues (Supplementary Table S1). Among these SNPs, 12 were non-synonymous single nucleotide variants, and 17 were genome-wide significant (*P* < 5.0 × 10^−8^) (Supplementary Table S1). Some of these m^6^A-SNPs showed eQTLs in different tissues or cells (Supplementary Table S2). Six of the 27 SNPs showed associations in both Asians and Europeans, including rs2305480 (*GSDMB*), rs1042308 (*HLA-DPA1*), rs3748816 (*MMEL1*), rs241449 (*TAP2*), rs2857433 (*TRIM10*), and rs757262 (*TRIM40*). For the associations between the eQTL m^6^A-SNPs and RA in Asians, only rs241449 (*TAP2*) and rs1042308 (*HLA-DPA1*) were significant at genome-wide scale.

A proportion of m^6^A-SNPs were missense mutations or variants which could influence transcription. The major function of missense mutation was changing the encoded amino acids. However, the functions of the missense m^6^A-SNPs should not be confined to amino acid change, but RNA processing. Studies have suggested that m^6^A-SNPs could also influence promoter activity, mRNA stability and subcellular localization of mRNAs and/or proteins (Shastry, 2009). The functions of m^6^A-SNPs in UTRs should not be confined to alter microRNA or transcription factor bindings either. Several significant m^6^A-SNPs in the MHC region also found to locate in transcription factor binding sites, DNase I hypersensitive sites or CpG islands (Figures S1–S7). The missense mutation rs3748816 in *MMEL1* (1p36.32) locates in a DNase I hypersensitive site and very closed to a CpG island (Figure S8).

### Differential expression analysis

For the above 24 genes, we compared mRNA expression signals in PBMCs of 26 RA cases and 17 controls and 3 published expression studies. In these 4 expression datasets, we found that 20 (17 in PBMCs) of them were differentially expressed in at least one study (*P* < 0.05) (Table 1, Supplementary Table S1). Among them, *MAPK13* and *HLA-DPB1* were differentially expressed in PBMCs and synovial tissues. There were 23 RA-associated m^6^A-SNPs in these 20 differential expression genes. *HLA-A* contained 2 (rs1136702 and rs3173419) and *HLA-C* contain 3 (rs1131123, rs35075694, and rs707908) RA-associated m^6^A-SNPs. Therefore, in total, we found 23 m^6^A-SNPs that formed 23 SNP-Gene-RA trios (Table 1). It means that these m^6^A-SNPs may affect RA risk through altering the expressions of local genes. Most of these m^6^A-SNPs showed eQTLs in multiple tissues or cells (Supplementary Table S2). We further searched for potential functionalities for these 23 m^6^A-SNPs in transcription regulation, such as altering protein binding. In RegulomeDB and HaploReg, we found that 10 of the 23 m^6^A-SNPs might alter the binding of one or more proteins and 16 might alter regulatory motifs in different cell types (Supplementary Table S2). However, the effects of the detected SNPs on m^6^A modification have not been validated by technical and biological experiments. Further experiments are needed to determine their functions in human cell lines.

**Table 1:** The identified 23 SNP-Gene-RA trios.

**SNP rsID**	**CHR**	**SNP position**^†^	**Mutation Type**^‡^	***P* value RA**	**Gene**	***P*****-value Differential expression**				***P*****-value eQTL**	
						**PBMCs**	**GSE15573**	**GSE17755**	**GSE1919**	**PBMCs**	**HaploReg**
**ASIANS**											
rs2076595	1	17396703	S	3.10E-06	*PADI2*	8.85E-03	–	6.23E-20	–	6.43E-06	2.19E-13
rs13978	6	26599043	UTR3	3.30E-11	*ABT1*	5.45E-04	–	–	–	1.98E-02	–
rs2857433	6	30120633	UTR3	4.90E-05	*TRIM10*	2.51E-04	–	–	–	1.65E-02	8.71E-24
rs241449	6	32796653	S	1.10E-17	*TAP2*	2.72E-02	–	6.03E-03	–	6.75E-09	9.81E-198
rs1042308	6	33036853	N	8.70E-11	*HLA-DPA1*	–	1.92E-04	2.78E-19	–	1.94E-08	1.18E-50
rs2071863	6	36107172	UTR3	2.20E-05	*MAPK13*	3.44E-05	3.52E-02	1.18E-06	3.78E-03	–	7.51E-15
rs9800580	6	149953981	S	3.50E-05	*KATNA1*	2.26E-03	3.00E-02	1.44E-02	–	–	3.64E-07
rs3924871	6	149983216	S	2.80E-05	*LATS1*	1.31E-03	–	1.02E-20	–	8.82E-04	6.29E-09
rs3763162	6	150140810	UTR3	1.80E-05	*LRP11*	–	–	2.88E-04	–	–	8.98E-09
rs2305480	17	38062196	N	8.80E-05	*GSDMB*	3.00E-03	–	–	–	1.26E-03	9.81E-198
**EUROPEANS**											
rs11545587	6	28891522	UTR5	2.10E-06	*TRIM27*	–	–	–	2.53E-02	–	4.75E-06
rs35771565	6	29012067	N	5.00E-09	*OR2W1*	–	–	5.38E-06	–	–	2.92E-06
rs1136702	6	29911092	N	1.50E-31	*HLA-A*	–	–	2.82E-07	–	2.93E-03	2.38E-07
rs3173419	6	29911119	N	5.20E-08	*HLA-A*	–	–	2.82E-07	–	–	1.38E-09
rs2857433	6	30120633	UTR3	1.50E-49	*TRIM10*	2.51E-04	–	–	–	1.65E-02	8.71E-24
rs9263697	6	31093581	UTR5	1.80E-11	*PSORS1C1*	–	–	2.34E-09	–	–	8.81E-17
rs130077	6	31122330	S	7.30E-10	*CCHCR1*	–	–	4.18E-04	–	–	3.05E-07
rs35075694	6	31236534	UTR3	1.90E-61	*HLA-C*	–	–	5.34E-06	2.52E-02	–	8.86E-16
rs707908	6	31238053	N	5.10E-10	*HLA-C*	–	–	5.34E-06	2.52E-02	–	1.22E-59
rs1131123	6	31239378	N	2.00E-37	*HLA-C*	–	–	5.34E-06	2.52E-02	–	1.08E-47
rs2227956	6	31778272	N	2.50E-49	*HSPA1L*	1.71E-03	–	–	–	–	5.06E-04
rs1033500	6	32307382	N	1.00E-250	*C6orf10*	2.58E-02	–	–	–	–	1.97E-06
rs241449	6	32796653	UTR3	6.50E-09	*TAP2*	–	–	6.03E-03	–	6.75E-09	9.81E-198
rs1042308	6	33036853	N	9.10E-78	*HLA-DPA1*	–	1.92E-04	2.78E-19	–	1.94E-08	1.18E-50
rs1042136	6	33048628	N	2.50E-34	*HLA-DPB1*	–	1.39E-02	4.84E-20	3.40E-03	2.61E-04	5.91E-18
rs923829	12	58174306	S	9.10E-06	*METTL21B*	3.34E-02	–	–	–	–	6.48E-30
rs2305480	17	38062196	N	5.00E-09	*GSDMB*	3.00E-03	–	–	–	–	9.81E-198

† *Assembly: GRCh37.p13*.

‡ *: N, non-synonymous; S, synonymous; UTR3, 3′-untranslated region; UTR5, 5′-untranslated region. CHR, Chromosome*.

As shown in Supplementary Table S3, CIBERSORT algorithm inferred the relative proportions of immune cells in PBMCs of the 26 RA cases and 17 controls. The PBMCs mainly contained monocytes (highest proportion), CD8 T cells, B cells, regulatory T cells (Tregs), and activated natural killer (NK) cells. Significantly (Bonferroni correction) lower proportions of B cells were observed in RA patients. Further we performed the differential expression analyses for the 17 genes in each cell subgroup of PBMCs. We found that the differences exist for 17 genes in B cells, 14 genes in NK cells, 4 genes (*C6orf10, HLA-A, TRIM10*, and *TRIM27*) in CD8 T cells, and only *TRIM39* in monocytes (Supplementary Table S3). The above results showed that the mean proportions of cell subgroups varied and were significantly different between RA cases and controls (e.g., B cells). Besides, the differences between the cell type components seemed to be associated with the number of significant genes (e.g., 17/17 in B cells; 14/17 in NK cells). These results were not beyond our expectation because PBMCs is a group of mixed cells, and the expression level of a gene specific to each cell types should be positively related to the proportion of the cell type. In addition, PBMCs is a group of immune associated cells, each of which may have cell type specific genes related to RA. Therefore, we believe that PBMCs could be commonly used as target cells for RA study, especially in initial discovery, because this would increase the possibility of finding a significant gene. Of course, the identified genes in PBMCs in initial discovery should be further investigated in cell subgroups. But on the other hand, we should realize that the use of PBMCs might also hide differences in gene expression if one cell type has increased and another one decreased expression of a gene.

In summary, the present study found many RA-associated m^6^A-SNPs which may play important roles in the pathogenesis of RA and demonstrated the potential functionality of these SNPs. The findings increase our understanding on the regulation patterns of SNP, and may provide new insight into the mechanism underlying the associations between SNPs and RA. Future studies are needed to confirm the m^6^A-SNP associations and to elucidate the mechanisms.

## Datasets availability

Data of RA GWAS was publicly available at http://plaza.umin.ac.jp/~yokada/datasource/software.htm. The m^6^A-SNPs data can be downloaded from the m6AVar database (http://m6avar.renlab.org/). The three public gene expression datasets, GSE15573, GSE17755, and GSE1919 can be downloaded from GEO database (https://www.ncbi.nlm.nih.gov/geo/). The raw data (excel file) supporting the conclusions of the eQTL analysis is included in the supplementary files.

## Author contributions

S-FL and Y-HZ conceived and designed the study. X-BM analyzed the data. X-BM wrote the paper.

### Conflict of interest statement

The authors declare that the research was conducted in the absence of any commercial or financial relationships that could be construed as a potential conflict of interest.
