# Supplementary material for: Genome-Wide Identification of N6-Methyladenosine (m6A) SNPs Associated With Rheumatoid Arthritis
Source: Front Genet. 2018 Aug 3;9:299. doi: 10.3389/fgene.2018.00299 (PMC6085591; doi:10.3389/fgene.2018.00299)
Supplement: Supplementary file 1 [file Data_Sheet_1.PDF]

Supplementary Table S1 m6A-SNPs showed association with RA at P < 0.0001

SNP rsID	CHR	SNP position†	Mutation Type	OR	OR_95%CI low	OR_95%CI up	P value RA	Gene	P value DE PBMCs	P value DE GSE15573	P value DE GSE17755	P value DE GSE1919	eQTL PBMCs	Beta PBMCs	P value eQTL HaploReg	m6A ID	m6A Position†	Distance	Confidence Level	m6A Function
Asians																				
rs3748816	1	2526746	nonsynonymous	1.11	1.06	1.17	4.90E-05	*MMEL1*							2.67E-32	m6A_ID_111730	2526743	3	Prediction:(Low)	Functional Loss
rs2076595	1	17396703	synonymous	0.89	0.85	0.93	3.10E-06	*PADI2*	8.85E-03		6.23E-20		6.43E-06	0.68	2.19E-13	m6A_ID_114224	1.7E+07	2	Prediction:(Low)	Functional Gain
rs4712960	6	25727265	synonymous	1.15	1.09	1.22	1.10E-06	*HIST1H2BA*					4.58E-02	0.15	3.06E-11	m6A_ID_189512	2.6E+07	29	Prediction:(Low)	Functional Loss
rs3734525	6	25779727	UTR3	0.69	0.61	0.77	1.80E-10	*SLC17A4*			1.61E-03					m6A_ID_189523	2.6E+07	2	Prediction:(Low)	Functional Gain
rs62625350	6	26094750	UTR3	1.48	1.32	1.66	1.30E-11	*HFE*	2.92E-04		4.55E-06	4.50E-02				m6A_ID_189591	2.6E+07	2	Prediction:(Low)	Functional Gain
**rs13978**	6	26599043	UTR3	1.47	1.31	1.64	3.30E-11	*ABT1*	5.45E-04				1.98E-02	1.21		m6A_ID_71029	2.7E+07	2	**PA-m6A-Seq:(High)**	Functional Loss
rs853678	6	28297313	nonsynonymous	1.17	1.1	1.25	1.60E-06	*ZSCAN31*								m6A_ID_71136	2.8E+07	0	MeRIP-Seq:(Medium)	Functional Loss
rs853678	6	28297313	nonsynonymous	1.17	1.1	1.25	1.60E-06	*ZSCAN31*								m6A_ID_71137	2.8E+07	0	MeRIP-Seq:(Medium)	Functional Loss
rs757262	6	30114955	nonsynonymous	1.14	1.07	1.22	3.20E-05	*TRIM40*					2.38E-02	-0.18	1.28E-27	m6A_ID_71378	3E+07	1	MeRIP-Seq:(Medium)	Functional Loss
rs2857433	6	30120633	UTR3	0.88	0.83	0.94	4.90E-05	*TRIM10*	2.51E-04				1.65E-02	-0.18	8.71E-24	m6A_ID_71382	3E+07	0	MeRIP-Seq:(Medium)	Functional Loss
rs16897791	6	30644359	UTR3	0.83	0.77	0.89	4.10E-07	*PPP1R18*	2.33E-05							m6A_ID_190274	3.1E+07	1	Prediction:(Low)	Functional Loss
rs2240803	6	30920957	UTR3	1.14	1.08	1.2	2.20E-07	*DPCR1*								m6A_ID_190410	3.1E+07	2	Prediction:(Low)	Functional Loss
**rs58892873**	6	31599360	synonymous	0.6	0.54	0.67	6.50E-22	*PRRC2A*	2.47E-02							m6A_ID_13970	3.2E+07	0	**miCLIP:(High)**	Functional Loss
rs928814	6	31631907	synonymous	0.59	0.53	0.66	1.20E-19	*GPANK1*	3.21E-02							m6A_ID_190604	3.2E+07	2	Prediction:(Low)	Functional Gain
rs2071282	6	32188943	nonsynonymous	2.04	1.9	2.19	2.50E-87	*NOTCH4*								m6A_ID_191138	3.2E+07	2	Prediction:(Low)	Functional Gain
rs367398	6	32191730	UTR5	1.24	1.18	1.3	4.70E-17	*NOTCH4*								m6A_ID_191141	3.2E+07	2	Prediction:(Low)	Functional Gain
rs241449	6	32796653	UTR3	1.25	1.19	1.31	1.10E-17	*TAP2*	2.72E-02		6.03E-03		6.75E-09	-1.02	9.81E-198	m6A_ID_191248	3.3E+07	24	Prediction:(Low)	Functional Gain
rs1042308	6	33036853	nonsynonymous	1.18	1.12	1.24	8.70E-11	*HLA-DPA1*		1.92E-04	2.78E-19		1.94E-08	-0.91	1.18E-50	m6A_ID_191327	3.3E+07	2	Prediction:(Low)	Functional Loss
rs1042308	6	33036853	nonsynonymous	1.18	1.12	1.24	8.70E-11	*HLA-DPA1*		1.92E-04	2.78E-19		1.94E-08	-0.91	1.18E-50	m6A_ID_191328	3.3E+07	2	Prediction:(Low)	Functional Loss
rs1042308	6	33036853	nonsynonymous	1.18	1.12	1.24	8.70E-11	*HLA-DPA1*		1.92E-04	2.78E-19		1.94E-08	-0.91	1.18E-50	m6A_ID_191330	3.3E+07	2	Prediction:(Low)	Functional Loss
rs2071863	6	36107172	UTR3	1.11	1.06	1.17	2.20E-05	*MAPK13*	3.44E-05	3.52E-02	1.18E-06	3.78E-03			7.51E-15	m6A_ID_192021	3.6E+07	22	Prediction:(Low)	Functional Loss
rs9800580	6	149953981	synonymous	0.89	0.84	0.94	3.50E-05	*KATNA1*	2.26E-03	3.00E-02	1.44E-02				3.64E-07	m6A_ID_198736	1.5E+08	2	Prediction:(Low)	Functional Loss
rs3924871	6	149983216	synonymous	0.89	0.84	0.94	2.80E-05	*LATS1*	1.31E-03		1.02E-20		8.82E-04	-0.68	6.29E-09	m6A_ID_602076	1.5E+08	1	miCLIP:(High)	Functional Loss
rs3763162	6	150140810	UTR3	0.89	0.84	0.94	1.80E-05	*LRP11*			2.88E-04				8.98E-09	m6A_ID_198771	1.5E+08	16	Prediction:(Low)	Functional Loss
rs2819419	14	105406238	nonsynonymous	1.12	1.06	1.18	6.00E-05	*AHNAK2*	1.36E-03		1.41E-25					m6A_ID_279828	1.1E+08	1	Prediction:(Low)	Functional Gain
rs4264326	14	105411700	nonsynonymous	1.12	1.06	1.18	7.90E-05	*AHNAK2*	1.36E-03		1.41E-25					m6A_ID_37504	1.1E+08	3	MeRIP-Seq:(Medium)	Functional Loss
rs11846918	14	105415469	nonsynonymous	1.13	1.06	1.2	7.90E-05	*AHNAK2*	1.36E-03		1.41E-25					m6A_ID_37579	1.1E+08	28	MeRIP-Seq:(Medium)	Functional Loss
rs2305480	17	38062196	nonsynonymous	1.12	1.06	1.18	8.80E-05	*GSDMB*	3.00E-03				1.26E-03	-0.39	9.81E-198	m6A_ID_307505	3.8E+07	2	Prediction:(Low)	Functional Gain
rs3746964	21	45705988	synonymous	0.89	0.83	0.94	9.30E-05	*AIRE*	3.66E-03							m6A_ID_344699	4.6E+07	23	Prediction:(Low)	Functional Gain
Europeans																				
rs3748816	1	2526746	nonsynonymous	1.09	1.06	1.13	2.80E-08	*MMEL1*							2.67E-32	m6A_ID_111730	2526743	3	Prediction:(Low)	Functional Loss
rs28411034	1	38276997	UTR3	0.91	0.87	0.95	4.00E-05	*MTF1*	7.70E-03	2.30E-03		2.30E-02				m6A_ID_117332	3.8E+07	1	Prediction:(Low)	Functional Loss
rs450630	6	28542424	synonymous	0.93	0.91	0.96	8.30E-06	*SCAND3*								m6A_ID_71153	2.9E+07	1	MeRIP-Seq:(Medium)	Functional Loss

rs11545587	6	28891522	UTR5	1.13	1.07	1.19	2.10E-06	*TRIM27*	1.69E-04			2.53E-02			4.75E-06	m6A_ID_71169	2.9E+07	23	MeRIP-Seq:(Medium)	Functional Loss
rs35771565	6	29012067	nonsynonymous	0.88	0.84	0.92	5.00E-09	*OR2W1*			5.38E-06				2.92E-06	m6A_ID_189967	2.9E+07	1	Prediction:(Low)	Functional Loss
rs7752270	6	29053998	UTR3	1.2	1.14	1.27	5.60E-11	*OR2B3*			1.38E-04					m6A_ID_189972	2.9E+07	17	Prediction:(Low)	Functional Loss
rs1136702	6	29911092	nonsynonymous	1.23	1.19	1.28	1.50E-31	*HLA-A*	8.44E-07		2.82E-07		2.93E-03	-0.28	2.38E-07	m6A_ID_190093	3E+07	2	Prediction:(Low)	Functional Gain
**rs1136702**	6	29911092	nonsynonymous	1.23	1.19	1.28	1.50E-31	*HLA-A*	8.44E-07		2.82E-07		2.93E-03	-0.28	2.38E-07	m6A_ID_71290	3E+07	2	**MeRIP-Seq:(Medium**	Functional Loss
rs3173419	6	29911119	nonsynonymous	1.1	1.06	1.14	5.20E-08	*HLA-A*	8.44E-07		2.82E-07				1.38E-09	m6A_ID_71301	3E+07	1	MeRIP-Seq:(Medium)	Functional Loss
rs3173419	6	29911119	nonsynonymous	1.1	1.06	1.14	5.20E-08	*HLA-A*	8.44E-07		2.82E-07					m6A_ID_71302	3E+07	1	MeRIP-Seq:(Medium)	Functional Loss
rs3173419	6	29911119	nonsynonymous	1.1	1.06	1.14	5.20E-08	*HLA-A*	8.44E-07		2.82E-07					m6A_ID_71317	3E+07	1	MeRIP-Seq:(Medium)	Functional Loss
rs41559916	6	29911296	nonsynonymous	1.3	1.21	1.39	7.50E-14	*HLA-A*	8.44E-07		2.82E-07					m6A_ID_190106	3E+07	2	Prediction:(Low)	Functional Gain
**rs757262**	6	30114955	nonsynonymous	1.3	1.25	1.34	1.60E-50	*TRIM40*							1.28E-27	m6A_ID_71378	3E+07	1	**MeRIP-Seq:(Medium**	Functional Loss
**rs2857433**	6	30120633	UTR3	0.78	0.75	0.8	1.50E-49	*TRIM10*	2.51E-04				1.65E-02	-0.18	8.71E-24	m6A_ID_71382	3E+07	0	**MeRIP-Seq:(Medium**	Functional Loss
**rs2074474**	6	30309508	synonymous	1.12	1.08	1.16	1.90E-09	*TRIM39,TRIM39-RPP21*					6.53E-05	-0.27	1.22E-08	m6A_ID_71424	3E+07	4	**MeRIP-Seq:(Medium**	Functional Loss
**rs2074474**	6	30309508	synonymous	1.12	1.08	1.16	1.90E-09	*TRIM39,TRIM39-RPP21*					6.53E-05	-0.27	1.22E-08	m6A_ID_71424	3E+07	4	**MeRIP-Seq:(Medium**	Functional Loss
rs12179536	6	30993590	nonsynonymous	0.83	0.8	0.86	4.30E-21	*MUC22*								m6A_ID_190418	3.1E+07	3	Prediction:(Low)	Functional Gain
rs9263697	6	31093581	UTR5	0.77	0.72	0.83	1.80E-11	*PSORS1C1*			2.34E-09				8.81E-17	m6A_ID_190437	3.1E+07	3	Prediction:(Low)	Functional Gain
rs130077	6	31122330	synonymous	1.13	1.09	1.18	7.30E-10	*CCHCR1*			4.18E-04				3.05E-07	m6A_ID_190457	3.1E+07	1	Prediction:(Low)	Functional Loss
rs35075694	6	31236534	UTR3	1.4	1.35	1.46	1.90E-61	*HLA-C*	2.44E-02		5.34E-06	2.52E-02			8.86E-16	m6A_ID_190474	3.1E+07	2	Prediction:(Low)	Functional Gain
rs707908	6	31238053	nonsynonymous	0.9	0.87	0.93	5.10E-10	*HLA-C*	2.44E-02		5.34E-06	2.52E-02			1.22E-59	m6A_ID_190475	3.1E+07	1	Prediction:(Low)	Functional Loss
rs707908	6	31238053	nonsynonymous	0.9	0.87	0.93	5.10E-10	*HLA-C*	2.44E-02		5.34E-06	2.52E-02				m6A_ID_190476	3.1E+07	1	Prediction:(Low)	Functional Loss
**rs1131123**	6	31239378	nonsynonymous	0.82	0.79	0.84	2.00E-37	*HLA-C*	2.44E-02		5.34E-06	2.52E-02			1.08E-47	m6A_ID_71553	3.1E+07	0	**MeRIP-Seq:(Medium**	Functional Loss
**rs1131123**	6	31239378	nonsynonymous	0.82	0.79	0.84	2.00E-37	*HLA-C*	2.44E-02		5.34E-06	2.52E-02			1.08E-47	m6A_ID_71554	3.1E+07	0	**MeRIP-Seq:(Medium**	Functional Loss
**rs1051791**	6	31378965	nonsynonymous	0.57	0.47	0.69	7.70E-09	*MICA*								m6A_ID_71565	3.1E+07	3	**MeRIP-Seq:(Medium**	Functional Loss
**rs1051791**	6	31378965	nonsynonymous	0.57	0.47	0.69	7.70E-09	*MICA*								m6A_ID_71566	3.1E+07	3	**MeRIP-Seq:(Medium**	Functional Loss
rs3219190	6	31497975	UTR3	0.66	0.63	0.69	1.90E-80	*MCCD1*								m6A_ID_190514	3.1E+07	12	Prediction:(Low)	Functional Loss
rs2736182	6	31583312	nonsynonymous	0.73	0.65	0.83	8.20E-07	*AIF1*	3.84E-04	8.37E-06		2.20E-03				m6A_ID_190540	3.2E+07	2	Prediction:(Low)	Functional Loss
**rs1046080**	6	31595882	nonsynonymous	1.45	1.4	1.5	6.10E-87	*PRRC2A*	2.47E-02							m6A_ID_13969	3.2E+07	1	**miCLIP:(High)**	Functional Loss
**rs2736158**	6	31600304	nonsynonymous	1.37	1.23	1.51	3.10E-09	*PRRC2A*	2.47E-02							m6A_ID_12250	3.2E+07	1	**miCLIP:(High)**	Functional Loss
rs453098	6	31691657	synonymous	0.66	0.62	0.71	4.10E-32	*C6orf25*	2.40E-02		1.87E-03					m6A_ID_190631	3.2E+07	2	Prediction:(Low)	Functional Loss
rs2227956	6	31778272	nonsynonymous	1.37	1.32	1.43	2.50E-49	*HSPA1L*	1.71E-03						5.06E-04	m6A_ID_190696	3.2E+07	3	Prediction:(Low)	Functional Gain
rs3911893	6	31935567	nonsynonymous	0.65	0.59	0.71	2.00E-20	*SKIV2L*	1.74E-02		8.89E-08					m6A_ID_190802	3.2E+07	1	Prediction:(Low)	Functional Loss
rs41270450	6	32017173	nonsynonymous	0.86	0.8	0.92	9.70E-06	*TNXB*	2.74E-06		1.24E-06					m6A_ID_190901	3.2E+07	26	Prediction:(Low)	Functional Loss
rs1802036	6	32136029	UTR3	1.53	1.39	1.67	3.30E-19	*AGPAT1*		3.55E-02						m6A_ID_191049	3.2E+07	17	Prediction:(Low)	Functional Gain
**rs11553430**	6	32136771	UTR3	1.81	1.67	1.97	3.20E-45	*AGPAT1*		3.55E-02						m6A_ID_71729	3.2E+07	1	**MeRIP-Seq:(Medium**	Functional Loss
rs8192576	6	32165300	synonymous	1.46	1.21	1.77	9.40E-05	*NOTCH4*								m6A_ID_191098	3.2E+07	18	Prediction:(Low)	Functional Gain
rs367398	6	32191730	UTR5	0.82	0.8	0.85	9.50E-29	*NOTCH4*								m6A_ID_191141	3.2E+07	2	Prediction:(Low)	Functional Gain

rs1033500	6	32307382	nonsynonymous	1.75	1.7	1.8	1.00E-250	*C6orf10*	2.58E-02						1.97E-06	m6A_ID_191157	3.2E+07	2	Prediction:(Low)	Functional Gain
rs2073045	6	32339548	UTR5	1.61	1.56	1.66	1.90E-185	*C6orf10*	2.58E-02							m6A_ID_191162	3.2E+07	19	Prediction:(Low)	Functional Gain
rs241449	6	32796653	UTR3	0.9	0.87	0.93	6.50E-09	*TAP2*	2.72E-02		6.03E-03		6.75E-09	-1.02	9.81E-198	m6A_ID_191248	3.3E+07	24	Prediction:(Low)	Functional Gain
rs1042308	6	33036853	nonsynonymous	1.52	1.45	1.59	9.10E-78	*HLA-DPA1*		1.92E-04	2.78E-19		1.94E-08	-0.91	1.18E-50	m6A_ID_191327	3.3E+07	2	Prediction:(Low)	Functional Loss
rs1042308	6	33036853	nonsynonymous	1.52	1.45	1.59	9.10E-78	*HLA-DPA1*		1.92E-04	2.78E-19		1.94E-08	-0.91	1.18E-50	m6A_ID_191328	3.3E+07	2	Prediction:(Low)	Functional Loss
rs1042308	6	33036853	nonsynonymous	1.52	1.45	1.59	9.10E-78	*HLA-DPA1*		1.92E-04	2.78E-19		1.94E-08	-0.91	1.18E-50	m6A_ID_191330	3.3E+07	2	Prediction:(Low)	Functional Loss
rs1042136	6	33048628	nonsynonymous	1.31	1.26	1.37	2.50E-34	*HLA-DPB1*	2.00E-02	1.39E-02	4.84E-20	3.40E-03	2.61E-04	-1.19	5.91E-18	m6A_ID_191338	3.3E+07	2	Prediction:(Low)	Functional Loss
rs2229785	6	33141161	synonymous	1.13	1.1	1.17	1.40E-13	*COL11A2*			2.72E-03					m6A_ID_191367	3.3E+07	12	Prediction:(Low)	Functional Gain
rs2072915	6	33162082	UTR3	1.09	1.05	1.12	2.10E-07	*RXRB*	4.25E-04							m6A_ID_71790	3.3E+07	0	MeRIP-Seq:(Medium)	Functional Loss
rs923829	12	58174306	synonymous	1.08	1.04	1.11	9.10E-06	*METTL21B*	3.34E-02						6.48E-30	m6A_ID_262633	5.8E+07	4	Prediction:(Low)	Functional Gain
rs2305480	17	38062196	nonsynonymous	1.09	1.06	1.13	5.00E-09	*GSDMB*	3.00E-03						9.81E-198	m6A_ID_307505	3.8E+07	2	Prediction:(Low)	Functional Gain
rs67631215	19	9024994	nonsynonymous	0.91	0.87	0.95	6.60E-05	*MUC16*			2.37E-05					m6A_ID_322972	9024971	23	Prediction:(Low)	Functional Loss
rs67631215	19	9024994	nonsynonymous	0.91	0.87	0.95	6.60E-05	*MUC16*			2.37E-05					m6A_ID_322973	9024971	23	Prediction:(Low)	Functional Loss
rs1059293	21	34809693	UTR3	0.94	0.91	0.97	3.60E-05	*IFNGR2*	6.36E-03							m6A_ID_343220	3.5E+07	4	Prediction:(Low)	Functional Gain

†Assembly: GRCh37.p13

CHR: Chromosome
