# Supplementary material for: Genome-Wide Identification of N6-Methyladenosine (m6A) SNPs Associated With Rheumatoid Arthritis
Source: Front Genet. 2018 Aug 3;9:299. doi: 10.3389/fgene.2018.00299 (PMC6085591; doi:10.3389/fgene.2018.00299)
Supplement: Supplementary file 2 [file Data_Sheet_2.PDF]

Supplementary Table S2 The potential regulatory functions for the identified m6A-SNPs

No.	SNP rsID	CHR	SNP position†	Gene	Mutation Type	P value (RA)	RegulomeDB score	Bound Protein	Regulatory motifs	Tissue	Correlated gene	P value eQTL	PMID
1	rs207659	1	17396703	*PADI2*	synonymous	3.10E-06	-	-	BDP1	Heart Left Ventricle	*PADI2*	1.21E-06	25954001
										Whole Blood	*PADI2*	2.19E-13	25954001
										PBMCs	*PADI2*	6.43E-06	-
2	rs13978	6	26599043	*ABT1*	UTR3	3.30E-11	5	-	-	PBMCs	*ABT1*	1.98E-02	-
3	rs285743	6	30120633	*TRIM10*	UTR3	4.90E-05	6	-	ERalpha-a, ERalpha-a, Foxa, HNF4, HNF4, PPAR_1, PPAR_3, RAR, RORalpha1_1, RXR::LXR, RXRA, RXRA, RXRA, RXRA, TR4	Whole Blood	*TRIM10*	8.71E-24	24013639
										PBMCs	*TRIM10*	1.65E-02	-
4	rs241449	6	32796653	*TAP2*	synonymous	1.10E-17	1f	-	Ascl2, NRSF, RXRA, Rad21	Adipose Subcutaneous	*TAP2*	1.01E-17	25954001
										Adipose Visceral Omentum	*TAP2*	9.99E-13	25954001
										Adrenal Gland	*TAP2*	4.23E-07	25954001
										Artery Aorta	*TAP2*	1.85E-20	25954001
										Artery Coronary	*TAP2*	1.83E-10	25954001
										Artery Tibial	*TAP2*	4.46E-26	25954001
										Brain Caudate basal ganglia	*TAP2*	1.02E-08	25954001
										Brain Cerebellum	*TAP2*	2.13E-12	25954001
										Brain Cortex	*TAP2*	5.54E-08	25954001
										Brain Frontal Cortex BA9	*TAP2*	7.73E-07	25954001
										Brain Nucleus accumbens basal ganglia	*TAP2*	2.99E-09	25954001
										Brain Putamen basal ganglia	*TAP2*	1.55E-07	25954001
										Breast Mammary Tissue	*TAP2*	2.50E-14	25954001
										Cells EBV-transformed lymphocytes	*TAP2*	4.67E-12	25954001
										Cells Transformed fibroblasts	*TAP2*	1.12E-17	25954001
										Colon Sigmoid	*TAP2*	1.76E-11	25954001
										Colon Transverse	*TAP2*	4.04E-12	25954001
										Esophagus Gastroesophageal Junction	*TAP2*	7.67E-14	25954001
										Esophagus Mucosa	*TAP2*	1.56E-19	25954001
										Esophagus Muscularis	*TAP2*	4.01E-26	25954001
										Heart Atrial Appendage	*TAP2*	5.62E-16	25954001
										Heart Left Ventricle	*TAP2*	1.47E-13	25954001
										Lung	*TAP2*	1.63E-16	25954001

										Muscle Skeletal	*TAP2*	6.24E-23	25954001
										Nerve Tibial	*TAP2*	1.14E-34	25954001
										Ovary	*TAP2*	4.56E-13	25954001
										Pancreas	*TAP2*	3.51E-17	25954001
										Skin Not Sun Exposed Suprapubic	*TAP2*	6.26E-11	25954001
										Skin Sun Exposed Lower leg	*TAP2*	9.19E-20	25954001
										Stomach	*TAP2*	4.80E-11	25954001
										Testis	*TAP2*	4.21E-06	25954001
										Thyroid	*TAP2*	1.80E-27	25954001
										Uterus	*TAP2*	3.07E-10	25954001
										Whole Blood	*TAP2*	8.57E-07	25954001
										Cerbellum	*TAP2*	1.13E-10	20485568
										FrontalCortex	*TAP2*	4.27E-09	20485568
										TemporalCortex	*TAP2*	1.27E-09	20485568
										Heart	*TAP2*	2.72E-09	
										Lymphoblastoid EUR genelevel	*TAP2*	1.68E-23	24037378
										Whole Blood	*TAP2*	7.43E-113	24013639
										Whole Blood	*TAP2*	9.81E-198	24013639
5	rs104230	6	33036853	*HLA-DPA1*	nonsynonymous	8.70E-11	4	POLR2A	-	Adipose Subcutaneous	*HLA-DPA1*	6.41E-42	25954001
										Adipose Visceral Omentum	*HLA-DPA1*	1.64E-21	25954001
										Artery Aorta	*HLA-DPA1*	2.69E-23	25954001
										Artery Coronary	*HLA-DPA1*	5.48E-15	25954001
										Artery Tibial	*HLA-DPA1*	4.91E-39	25954001
										Brain Caudate basal ganglia	*HLA-DPA1*	2.35E-11	25954001
										Brain Cerebellar Hemisphere	*HLA-DPA1*	1.57E-11	25954001
										Brain Cerebellum	*HLA-DPA1*	5.54E-15	25954001
										Brain Cortex	*HLA-DPA1*	1.18E-07	25954001
										Brain Frontal Cortex BA9	*HLA-DPA1*	4.74E-07	25954001
										Brain Hippocampus	*HLA-DPA1*	1.01E-09	25954001
										Brain Hypothalamus	*HLA-DPA1*	2.20E-10	25954001
										Brain Nucleus accumbens basal ganglia	*HLA-DPA1*	3.97E-11	25954001
										Brain Putamen basal ganglia	*HLA-DPA1*	1.85E-09	25954001
										Breast Mammary Tissue	*HLA-DPA1*	6.68E-26	25954001
										Cells EBV-transformed lymphocytes	*HLA-DPA1*	9.82E-15	25954001
										Cells Transformed fibroblasts	*HLA-DPA1*	8.10E-14	25954001

										Colon Sigmoid	*HLA-DPA1*	4.67E-11	25954001
										Colon Transverse	*HLA-DPA1*	9.51E-16	25954001
										Esophagus Gastroesophageal Junction	*HLA-DPA1*	1.02E-13	25954001
										Esophagus Mucosa	*HLA-DPA1*	8.15E-28	25954001
										Esophagus Muscularis	*HLA-DPA1*	1.24E-36	25954001
										Heart Atrial Appendage	*HLA-DPA1*	3.20E-18	25954001
										Heart Left Ventricle	*HLA-DPA1*	1.71E-18	25954001
										Liver	*HLA-DPA1*	4.54E-08	25954001
										Lung	*HLA-DPA1*	4.43E-54	25954001
										Muscle Skeletal	*HLA-DPA1*	1.18E-50	25954001
										Nerve Tibial	*HLA-DPA1*	6.78E-42	25954001
										Pancreas	*HLA-DPA1*	8.21E-17	25954001
										Pituitary	*HLA-DPA1*	2.08E-08	25954001
										Prostate	*HLA-DPA1*	6.89E-08	25954001
										Skin Not Sun Exposed Suprapubic	*HLA-DPA1*	4.74E-30	25954001
										Skin Sun Exposed Lower leg	*HLA-DPA1*	1.26E-60	25954001
										Spleen	*HLA-DPA1*	5.22E-13	25954001
										Stomach	*HLA-DPA1*	1.91E-20	25954001
										Testis	*HLA-DPA1*	2.79E-23	25954001
										Thyroid	*HLA-DPA1*	5.82E-47	25954001
										Uterus	*HLA-DPA1*	2.36E-08	25954001
										Whole Blood	*HLA-DPA1*	5.69E-27	25954001
6	rs207186	6	36107172	*MAPK13*	UTR3	2.20E-05	4	CTCF	ELF1, Rad21, Znf143	Cells Transformed fibroblasts	*MAPK13*	2.07E-08	25954001
										Esophagus Mucosa	*MAPK13*	7.51E-15	25954001
										Pancreas	*MAPK13*	3.37E-07	25954001
										Skin Not Sun Exposed Suprapubic	*MAPK13*	2.36E-07	25954001
										Skin Sun Exposed Lower leg	*MAPK13*	7.73E-09	25954001
										Testis	*MAPK13*	5.31E-06	25954001
										Thyroid	*MAPK13*	3.46E-06	25954001
7	rs980058	6	149953981	*KATNA1*	synonymous	3.50E-05	-	-	NRSF, Smad3_2, Smad_1	Muscle Skeletal	*KATNA1*	3.64E-07	25954001
8	rs392487	6	149983216	*LATS1*	synonymous	2.80E-05	6	-	Barx1, Barx2, Bsx, HNF6, Sox_10, Sox_14, Sox_16, Sox_18, Sox_19, Sox_2, Sox_3, Sox_9	Brain Cerebellum	*LATS1*	6.29E-09	25954001
										Nerve Tibial	*LATS1*	2.87E-06	25954001

9	rs376316	6	150140810	*LRP11*	UTR3	1.80E-05	6	-	Foxd3, Foxo_2, IRC900814, Sox_15, Sox_16, Sox_18, Sox_19, Sox_9, Zfp105	Skin Sun Exposed Lower leg	*LRP11*	8.98E-09	25954001
										Testis	*LRP11*	1.11E-06	25954001
10	rs115455	6	28891522	*TRIM27*		2.10E-06	4	POLR2A, PHF8, MAX, MYC, ATF2, RBBP5, SAP30, YY1, CTCF, E2F6, SIN3A, STAT5A, TAF1, UBTF, MXI1, CHD2, PML, KDM5B, NR3C1, CCNT2, ELK4, HMGN3	P-2, CTCFL, HEY1, HNF4, NRSF, RXRA, SP1, TFII-I	Skin Sun Exposed Lower leg	*TRIM27*	4.75E-06	25954001
11	rs357715	6	29012067	*OR2W1*	nonsynonymous	5.00E-09	-	-	AP-1, HDAC2	Testis	*OR2W1*	2.92E-06	25954001
12	rs113670	6	29911092	*HLA-A*	nonsynonymous	1.50E-31	4	POLR2A	-	Artery Tibial	*HLA-A*	2.38E-07	25954001
										Testis	*HLA-A*	6.13E-06	25954001
13	rs317341	6	29911119	*HLA-A*	nonsynonymous	5.20E-08	4	POLR2A	-	Artery Tibial	*HLA-A*	1.38E-09	25954001
										Cells EBV-transformed lymphocytes	*HLA-A*	7.49E-07	25954001
										Cells Transformed fibroblasts	*HLA-A*	4.30E-07	25954001
										Thyroid	*HLA-A*	1.24E-06	25954001
14	rs926369	6	31093581	*PSORS1C1*	UTR5	1.80E-11	5	-	-	Adipose Subcutaneous	*PSORS1C1*	3.56E-07	25954001
										Adrenal Gland	*PSORS1C1*	3.00E-06	25954001
										Artery Aorta	*PSORS1C1*	7.43E-12	25954001
										Artery Coronary	*PSORS1C1*	1.42E-06	25954001
										Artery Tibial	*PSORS1C1*	3.38E-13	25954001
										Cells Transformed fibroblasts	*PSORS1C1*	1.25E-10	25954001
										Colon Sigmoid	*PSORS1C1*	1.81E-11	25954001
										Colon Transverse	*PSORS1C1*	2.22E-07	25954001
										Esophagus Gastroesophageal Junction	*PSORS1C1*	5.29E-08	25954001
										Esophagus Muscularis	*PSORS1C1*	5.80E-12	25954001
										Heart Left Ventricle	*PSORS1C1*	5.00E-06	25954001
										Lung	*PSORS1C1*	8.81E-17	25954001
										Skin Not Sun Exposed Suprapubic	*PSORS1C1*	2.26E-07	25954001
										Skin Sun Exposed Lower leg	*PSORS1C1*	7.49E-13	25954001
										Thyroid	*PSORS1C1*	3.23E-14	25954001

15	rs130077	6	31122330	*CCHCR1*	synonymous	7.30E-10	6	-	AP-3, Hoxa5_1, PU.1	Lung	*CCHCR1*	3.05E-07	25954001
16	rs350756	6	31236534	*HLA-C*	UTR3	1.90E-61	5	POLR2A, POU2F2	Hsf	Adipose Subcutaneous	*HLA-C*	6.62E-12	25954001
										Artery Aorta	*HLA-C*	1.12E-09	25954001
										Artery Coronary	*HLA-C*	1.43E-06	25954001
										Artery Tibial	*HLA-C*	1.75E-08	25954001
										Brain Nucleus accumbens basal ganglia	*HLA-C*	6.78E-08	25954001
										Cells Transformed fibroblasts	*HLA-C*	1.43E-07	25954001
										Colon Transverse	*HLA-C*	1.03E-08	25954001
										Esophagus Mucosa	*HLA-C*	7.07E-09	25954001
										Esophagus Muscularis	*HLA-C*	2.75E-06	25954001
										Heart Atrial Appendage	*HLA-C*	3.76E-08	25954001
										Lung	*HLA-C*	1.11E-08	25954001
										Muscle Skeletal	*HLA-C*	2.18E-11	25954001
										Nerve Tibial	*HLA-C*	4.37E-08	25954001
										Skin Not Sun Exposed Suprapubic	*HLA-C*	1.03E-06	25954001
										Skin Sun Exposed Lower leg	*HLA-C*	1.82E-08	25954001
										Testis	*HLA-C*	3.06E-06	25954001
										Thyroid	*HLA-C*	7.51E-12	25954001
										Whole Blood	*HLA-C*	8.86E-16	25954001
17	rs707908	6	31238053	*HLA-C*	nonsynonymous	5.10E-10	5	POLR2A	-	Adipose Subcutaneous	*HLA-C*	4.74E-57	25954001
										Adipose Visceral Omentum	*HLA-C*	1.84E-32	25954001
										Adrenal Gland	*HLA-C*	2.78E-19	25954001
										Artery Aorta	*HLA-C*	1.69E-44	25954001
										Artery Coronary	*HLA-C*	5.13E-20	25954001
										Artery Tibial	*HLA-C*	3.14E-64	25954001
										Brain Anterior cingulate cortex BA24	*HLA-C*	1.81E-15	25954001
										Brain Caudate basal ganglia	*HLA-C*	5.17E-19	25954001
										Brain Cerebellar Hemisphere	*HLA-C*	1.37E-18	25954001
										Brain Cerebellum	*HLA-C*	1.34E-25	25954001
										Brain Cortex	*HLA-C*	9.92E-17	25954001
										Brain Frontal Cortex BA9	*HLA-C*	4.92E-13	25954001
										Brain Hippocampus	*HLA-C*	1.28E-17	25954001
										Brain Hypothalamus	*HLA-C*	6.64E-18	25954001
										Brain Nucleus accumbens basal ganglia	*HLA-C*	9.10E-17	25954001
										Brain Putamen basal ganglia	*HLA-C*	2.77E-14	25954001

										Breast Mammary Tissue	*HLA-C*	3.11E-34	25954001
										Cells EBV-transformed lymphocytes	*HLA-C*	1.90E-24	25954001
										Cells Transformed fibroblasts	*HLA-C*	4.72E-67	25954001
										Colon Sigmoid	*HLA-C*	5.48E-26	25954001
										Colon Transverse	*HLA-C*	2.49E-36	25954001
										Esophagus Gastroesophageal Junction	*HLA-C*	3.18E-26	25954001
										Esophagus Mucosa	*HLA-C*	1.73E-48	25954001
										Esophagus Muscularis	*HLA-C*	4.76E-45	25954001
										Heart Atrial Appendage	*HLA-C*	3.36E-22	25954001
										Heart Left Ventricle	*HLA-C*	2.59E-37	25954001
										Liver	*HLA-C*	1.58E-24	25954001
										Lung	*HLA-C*	1.34E-46	25954001
										Muscle Skeletal	*HLA-C*	4.83E-75	25954001
										Nerve Tibial	*HLA-C*	2.85E-44	25954001
										Ovary	*HLA-C*	6.24E-15	25954001
										Pancreas	*HLA-C*	4.59E-23	25954001
										Pituitary	*HLA-C*	3.36E-18	25954001
										Prostate	*HLA-C*	1.29E-12	25954001
										Skin Not Sun Exposed Suprapubic	*HLA-C*	3.48E-42	25954001
										Skin Sun Exposed Lower leg	*HLA-C*	1.22E-59	25954001
										Small Intestine Terminal Ileum	*HLA-C*	4.01E-11	25954001
										Spleen	*HLA-C*	1.07E-14	25954001
										Stomach	*HLA-C*	6.45E-31	25954001
										Testis	*HLA-C*	2.24E-32	25954001
										Thyroid	*HLA-C*	8.40E-50	25954001
										Uterus	*HLA-C*	9.46E-10	25954001
										Vagina	*HLA-C*	9.07E-11	25954001
										Whole Blood	*HLA-C*	1.39E-44	25954001
18	rs113112	6	31239378	*HLA-C*	nonsynonymous	2.00E-37	4	POLR2A, CTCF, NFKB1,	AP-1, FXR_1, GR	Adipose Subcutaneous	*HLA-C*	2.39E-42	25954001
										Adipose Visceral Omentum	*HLA-C*	3.55E-21	25954001
										Adrenal Gland	*HLA-C*	2.64E-12	25954001
										Artery Aorta	*HLA-C*	2.57E-25	25954001
										Artery Coronary	*HLA-C*	3.91E-10	25954001
										Artery Tibial	*HLA-C*	5.08E-33	25954001

Brain Anterior cingulate cortex BA24	*HLA-C*	1.96E-12	25954001
Brain Caudate basal ganglia	*HLA-C*	2.98E-13	25954001
Brain Cerebellar Hemisphere	*HLA-C*	8.76E-09	25954001
Brain Cerebellum	*HLA-C*	4.74E-14	25954001
Brain Cortex	*HLA-C*	5.04E-19	25954001
Brain Frontal Cortex BA9	*HLA-C*	5.16E-12	25954001
Brain Hippocampus	*HLA-C*	1.53E-11	25954001
Brain Hypothalamus	*HLA-C*	1.57E-15	25954001
Brain Nucleus accumbens basal ganglia	*HLA-C*	1.27E-12	25954001
Brain Putamen basal ganglia	*HLA-C*	6.96E-11	25954001
Breast Mammary Tissue	*HLA-C*	3.50E-24	25954001
Cells EBV-transformed lymphocytes	*HLA-C*	1.69E-16	25954001
Cells Transformed fibroblasts	*HLA-C*	2.39E-26	25954001
Colon Sigmoid	*HLA-C*	6.66E-12	25954001
Colon Transverse	*HLA-C*	1.41E-18	25954001
Esophagus Gastroesophageal Junction	*HLA-C*	4.01E-15	25954001
Esophagus Mucosa	*HLA-C*	3.41E-25	25954001
Esophagus Muscularis	*HLA-C*	1.13E-26	25954001
Heart Atrial Appendage	*HLA-C*	1.03E-17	25954001
Heart Left Ventricle	*HLA-C*	1.09E-21	25954001
Liver	*HLA-C*	5.91E-15	25954001
Lung	*HLA-C*	1.02E-35	25954001
Muscle Skeletal	*HLA-C*	7.77E-49	25954001
Nerve Tibial	*HLA-C*	5.67E-38	25954001
Ovary	*HLA-C*	1.30E-13	25954001
Pancreas	*HLA-C*	6.32E-14	25954001
Pituitary	*HLA-C*	1.62E-07	25954001
Skin Not Sun Exposed Suprapubic	*HLA-C*	1.47E-20	25954001
Skin Sun Exposed Lower leg	*HLA-C*	1.97E-33	25954001
Small Intestine Terminal Ileum	*HLA-C*	4.50E-09	25954001
Spleen	*HLA-C*	7.11E-12	25954001
Stomach	*HLA-C*	1.30E-16	25954001
Testis	*HLA-C*	4.10E-12	25954001
Thyroid	*HLA-C*	1.26E-31	25954001
Uterus	*HLA-C*	3.14E-08	25954001

										Vagina	*HLA-C*	2.16E-10	25954001
										Whole Blood	*HLA-C*	1.08E-47	25954001
19	rs222795	6	31778272	*HSPA1L*	nonsynonymous	2.50E-49	1f	ESR1	Sin3Ak-20	Whole Blood	*HSPA1L*	5.06E-04	24013639
20	rs103350	6	32307382	*C6orf10*	nonsynonymous	1.00E-250	-	-	Ehf, Elf3, Elf5, p3000, PU.1	Testis	*C6orf10*	1.97E-06	25954001
21	rs104213	6	33048628	*HLA-DPB1*	nonsynonymous	2.50E-34	3a	POLR2A, BCL3, GABPB1, ZNF263, ELF1, FOXP2,	-	Adipose Subcutaneous	*HLA-DPB1*	8.78E-11	25954001
										Artery Aorta	*HLA-DPB1*	1.01E-13	25954001
										Artery Coronary	*HLA-DPB1*	6.35E-08	25954001
										Artery Tibial	*HLA-DPB1*	1.60E-15	25954001
										Breast Mammary Tissue	*HLA-DPB1*	9.74E-07	25954001
										Cells Transformed fibroblasts	*HLA-DPB1*	4.69E-06	25954001
										Colon Transverse	*HLA-DPB1*	2.10E-09	25954001
										Esophagus Gastroesophageal Junction	*HLA-DPB1*	2.13E-08	25954001
										Esophagus Mucosa	*HLA-DPB1*	8.30E-12	25954001
										Esophagus Muscularis	*HLA-DPB1*	2.29E-17	25954001
										Heart Atrial Appendage	*HLA-DPB1*	7.88E-07	25954001
										Heart Left Ventricle	*HLA-DPB1*	6.45E-10	25954001
										Lung	*HLA-DPB1*	1.97E-15	25954001
										Muscle Skeletal	*HLA-DPB1*	6.05E-15	25954001
										Nerve Tibial	*HLA-DPB1*	2.20E-14	25954001
										Pancreas	*HLA-DPB1*	5.73E-08	25954001
										Skin Not Sun Exposed Suprapubic	*HLA-DPB1*	2.20E-06	25954001
										Skin Sun Exposed Lower leg	*HLA-DPB1*	1.32E-16	25954001
										Spleen	*HLA-DPB1*	1.49E-07	25954001
										Stomach	*HLA-DPB1*	2.05E-07	25954001
										Thyroid	*HLA-DPB1*	1.28E-17	25954001
										Whole Blood	*HLA-DPB1*	5.91E-18	25954001
22	rs923829	12	58174306	*METTL21B*	synonymous	9.10E-06	6	-	GR,Myc	Adipose Subcutaneous	*METTL21B*	6.48E-30	25954001
										Adipose Visceral Omentum	*METTL21B*	1.30E-17	25954001
										Adrenal Gland	*METTL21B*	1.15E-13	25954001
										Artery Aorta	*METTL21B*	4.05E-17	25954001
										Artery Coronary	*METTL21B*	3.54E-07	25954001
										Artery Tibial	*METTL21B*	9.41E-18	25954001
										Brain Anterior cingulate cortex BA24	*METTL21B*	5.86E-07	25954001
										Brain Caudate basal ganglia	*METTL21B*	1.09E-12	25954001

										Brain Cerebellar Hemisphere	*METTL21B*	6.24E-16	25954001
										Brain Cerebellum	*METTL21B*	1.35E-20	25954001
										Brain Cortex	*METTL21B*	5.70E-15	25954001
										Brain Frontal Cortex BA9	*METTL21B*	2.21E-09	25954001
										Brain Hippocampus	*METTL21B*	2.38E-09	25954001
										Brain Hypothalamus	*METTL21B*	9.96E-10	25954001
										Brain Nucleus accumbens basal ganglia	*METTL21B*	4.59E-18	25954001
										Brain Putamen basal ganglia	*METTL21B*	4.08E-11	25954001
										Breast Mammary Tissue	*METTL21B*	6.06E-09	25954001
										Cells EBV-transformed lymphocytes	*METTL21B*	1.09E-13	25954001
										Cells Transformed fibroblasts	*METTL21B*	2.74E-50	25954001
										Colon Sigmoid	*METTL21B*	6.16E-12	25954001
										Colon Transverse	*METTL21B*	7.31E-17	25954001
										Esophagus Gastroesophageal Junction	*METTL21B*	3.67E-13	25954001
										Esophagus Mucosa	*METTL21B*	6.57E-17	25954001
										Esophagus Muscularis	*METTL21B*	4.50E-24	25954001
										Heart Atrial Appendage	*METTL21B*	3.91E-11	25954001
										Heart Left Ventricle	*METTL21B*	3.86E-07	25954001
										Lung	*METTL21B*	5.53E-18	25954001
										Muscle Skeletal	*METTL21B*	1.02E-13	25954001
										Nerve Tibial	*METTL21B*	4.25E-25	25954001
										Pancreas	*METTL21B*	6.60E-13	25954001
										Pituitary	*METTL21B*	4.24E-07	25954001
										Skin Not Sun Exposed Suprapubic	*METTL21B*	1.28E-09	25954001
										Skin Sun Exposed Lower leg	*METTL21B*	5.40E-25	25954001
										Spleen	*METTL21B*	2.67E-08	25954001
										Stomach	*METTL21B*	5.91E-15	25954001
										Testis	*METTL21B*	1.91E-05	25954001
										Whole Blood	*METTL21B*	1.89E-25	25954001
23	rs230548	17	38062196	*GSDMB*	nonsynonymous	5.00E-09	1f	-	BAF155, TAL1	Cells EBV-transformed lymphocytes	*GSDMB*	3.92E-06	25954001
										Colon Transverse	*GSDMB*	3.98E-07	25954001
										Small Intestine Terminal Ileum	*GSDMB*	2.22E-07	25954001
										Spleen	*GSDMB*	8.15E-10	25954001
										Whole Blood	*GSDMB*	3.95E-22	25954001
										Whole Blood	*GSDMB*	9.81E-198	24013639
