# Supplementary material for: Genome-Wide Identification of N6-Methyladenosine (m6A) SNPs Associated With Rheumatoid Arthritis
Source: Front Genet. 2018 Aug 3;9:299. doi: 10.3389/fgene.2018.00299 (PMC6085591; doi:10.3389/fgene.2018.00299)
Supplement: Supplementary file 3 [file Data_Sheet_3.PDF]

Supplementary Table S3 Differential expression of the 17 genes in major cell types of PBMCs

	Proportion (Mean ±SD)			*P* values of differential expression*																
Subtype	Case	Control	*P*	*ABT1*	*C6orf10*	*CCHCR1*	*GSDMB*	*HLA-A*	*HLA-C*	*HLA-DPB1*	*HSPA1L*	*KATNA1*	*LATS1*	*MAPK13*	*METTL21B*	*PADI2*	*TAP2*	*TRIM10*	*TRIM27*	*TRIM39*
B cells	0.03 ±0.02	0.06 ±0.03	0.002	0.007	0.002	0.003	0.013	0.002	0.005	0.003	0.004	0.012	0.010	0.002	0.007	0.008	0.006	0.001	0.002	0.020
T cells CD8	0.28 ±0.07	0.32 ±0.07	0.136	0.643	0.046	0.109	0.557	0.028	0.224	0.080	0.250	0.617	0.688	0.070	0.361	0.520	0.305	0.012	0.049	0.987
Tregs	0.05 ±0.03	0.06 ±0.04	0.733	0.792	0.671	0.850	0.858	0.678	0.998	0.809	0.994	0.649	0.611	0.775	0.962	0.904	0.995	0.537	0.745	0.536
NK cells	0.05 ±0.03	0.07 ±0.04	0.041	0.051	0.005	0.012	0.045	0.006	0.018	0.010	0.021	0.047	0.054	0.008	0.029	0.034	0.019	0.005	0.008	0.096
Monocytes	0.39 ±0.10	0.38 ±0.08	0.728	0.129	0.493	0.866	0.218	0.530	0.553	0.980	0.442	0.155	0.089	0.880	0.339	0.218	0.461	0.450	0.791	0.045

P values less than 0.05 were marked in red.

*: Five genes were differentially expressed in B cells after adjusted for B cell proportions,
including GSDMB (*P* = 0.0217), HLA-A (*P* = 0.0244), TRIM10 (*P* = 0.0323), TRIM27 (*P* = 0.0382) and TRIM39 (*P* = 0.0255);
Ten genes were differentially expressed in NK cells after adjusted for NK cell proportions,
including ABT1 (*P* = 0.00447), GSDMB (*P* = 0.00378), HLA-A (*P* = 0.00307), HSPA1L (*P* = 0.029), KATNA1 (*P* = 0.0418),
LATS1 (*P* = 0.0377), MAPK13 (*P* = 0.0055), TRIM10 (*P* = 0.0229), TRIM27 (*P* = 0.022) and TRIM39 (*P* = 0.0189).
