# Supplementary material for: Genome-Wide Identification of N6-Methyladenosine (m6A) SNPs Associated With Rheumatoid Arthritis
Source: Front Genet. 2018 Aug 3;9:299. doi: 10.3389/fgene.2018.00299 (PMC6085591; doi:10.3389/fgene.2018.00299)
Supplement: Supplementary file 5 [file Image_1.PDF]

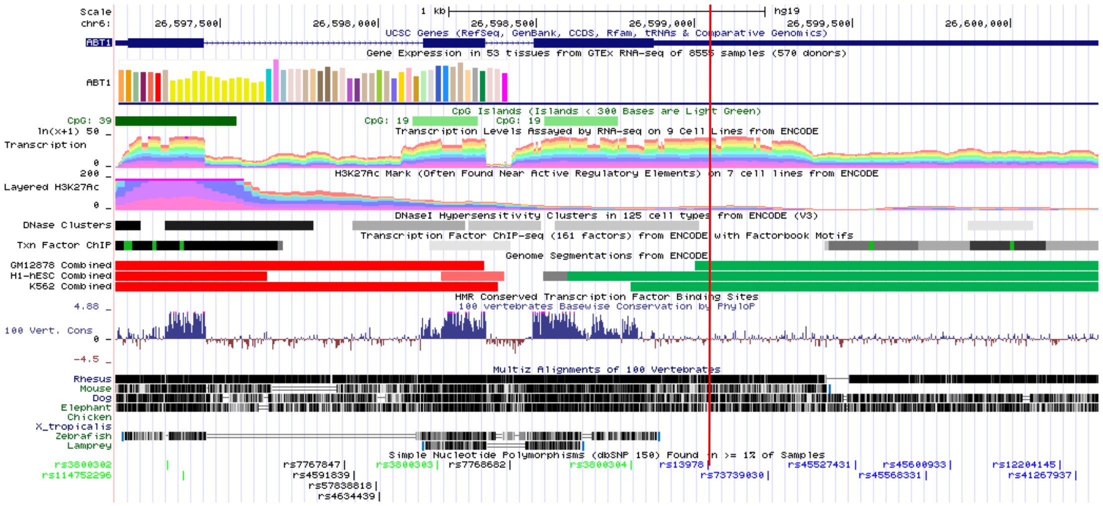

Figure S1 The genomic region of rs13978 in *ABT1* gene

SNP rs13978 locates in the 3′-UTR of *ABT1*. It locates very closed to a DNase I hypersensitive site and a CpG island.

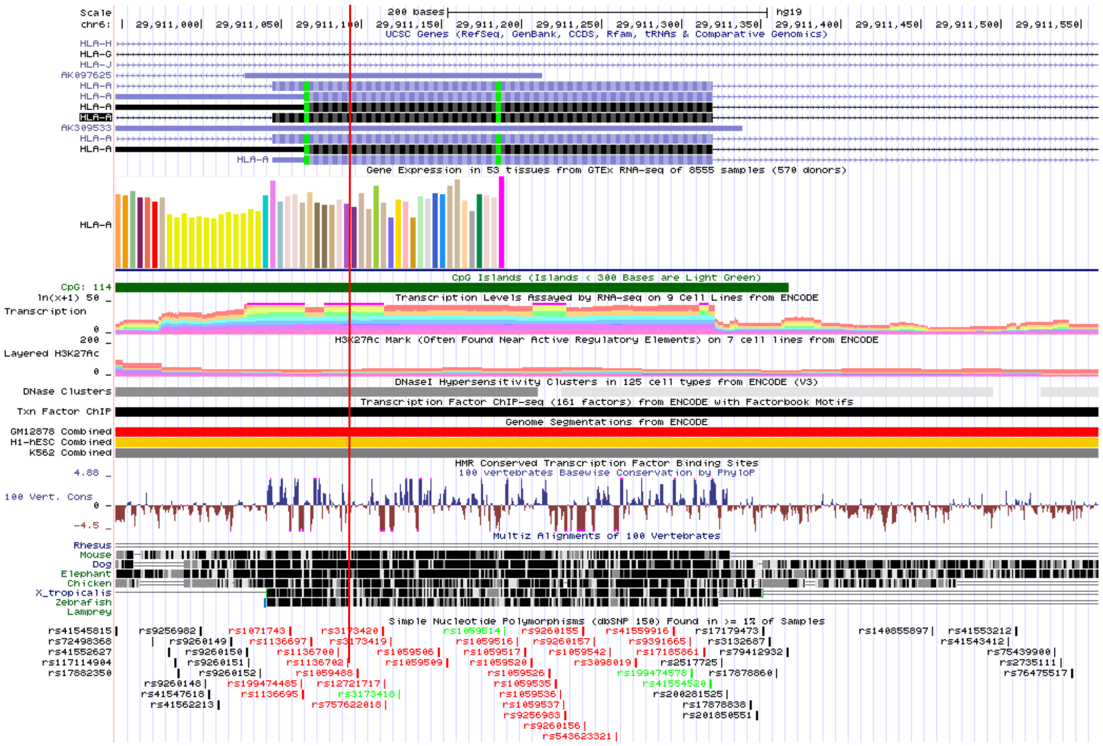

Figure S2 The genomic region of rs1136702 in *HLA-A* gene

This region shows very high conservation, transcription level and DNaseI hypersensitivity. The missense SNP rs1136702 locates in exon 3 of *HLA-A*. It locates in a DNase I hypersensitive site and very closed to a CpG island.

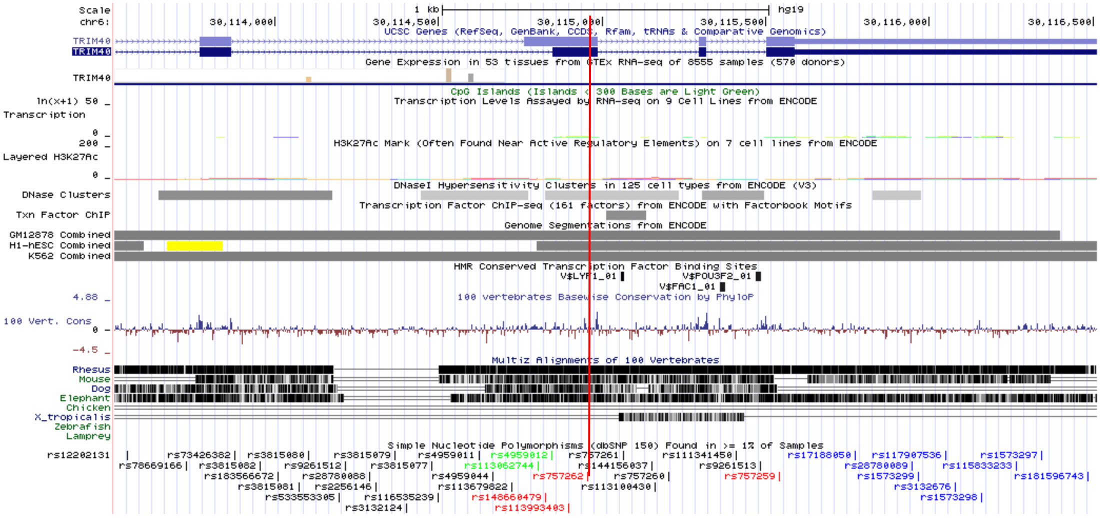

Figure S3 The genomic region of rs757262 in *TRIM40* gene

The missense SNP rs757262 locates in exon 3 of *TRIM40*. It locates in a DNase I hypersensitive site and very closed to a transcription factor binding site.

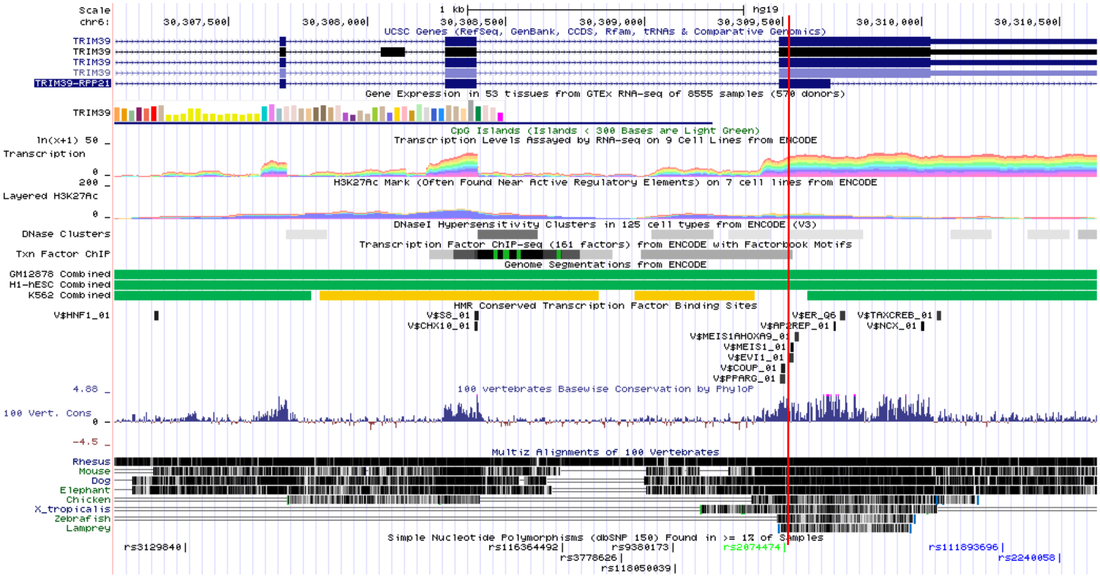

Figure S4 The genomic region of rs2074474 in *TRIM39* gene

This region shows very high conservation, transcription level and DNaseI hypersensitivity. The synonymous SNP rs2074474 locates in exon 6 of *TRIM39*. It locates in a DNase I hypersensitive site and transcription factor binding sites.

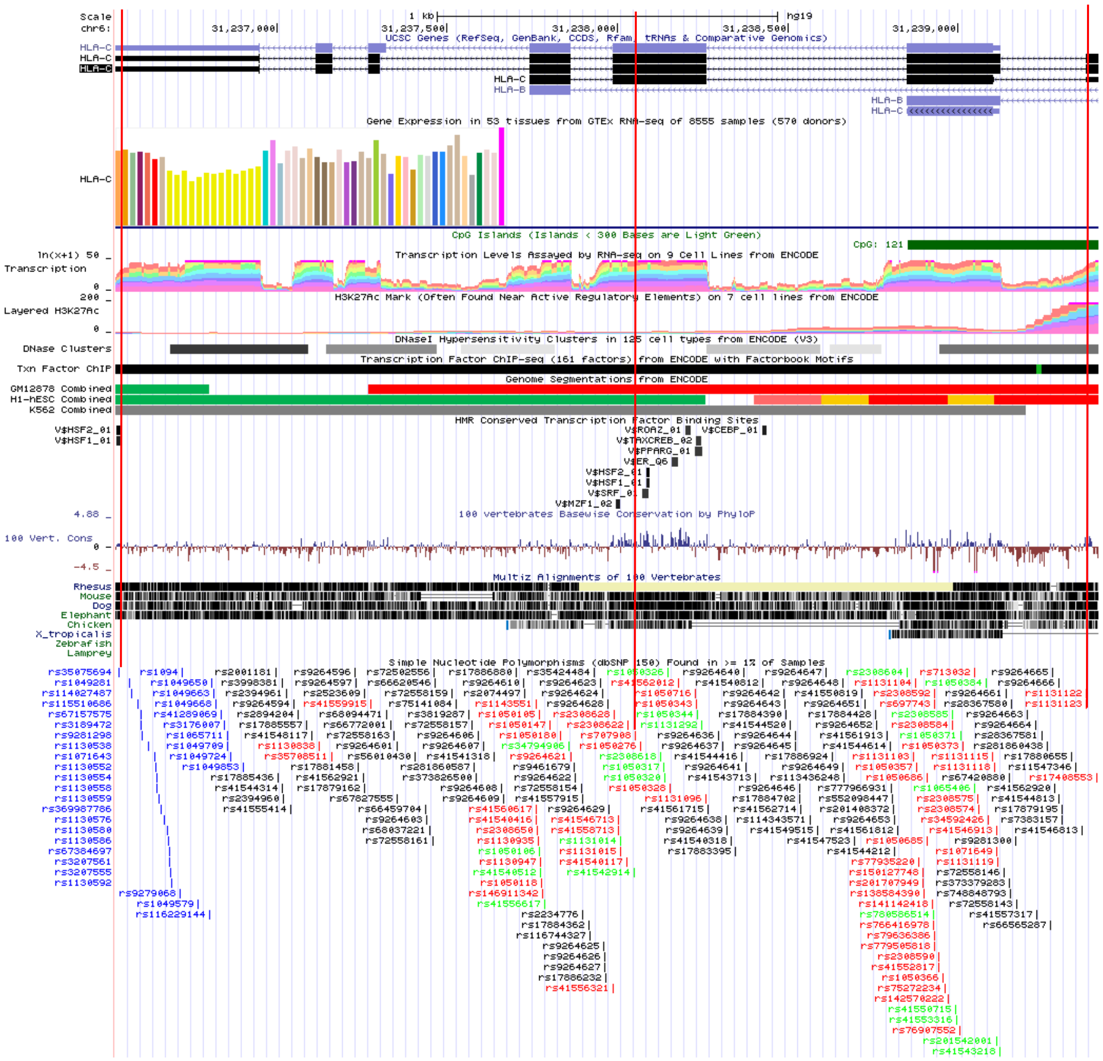

Figure S5 The genomic region of the *HLA-C* gene

This region shows very high conservation, transcription level and DNaseI hypersensitivity. SNP rs35075694 and the missense SNPs rs707908 and rs1131123 locate in the 3′-UTR and exons 4 and 2 of *HLA-C*. SNP rs35075694 and rs707908 locate in transcription factor binding sites. SNP rs1131123 locates very closed to a DNase I hypersensitive site and a CpG island.

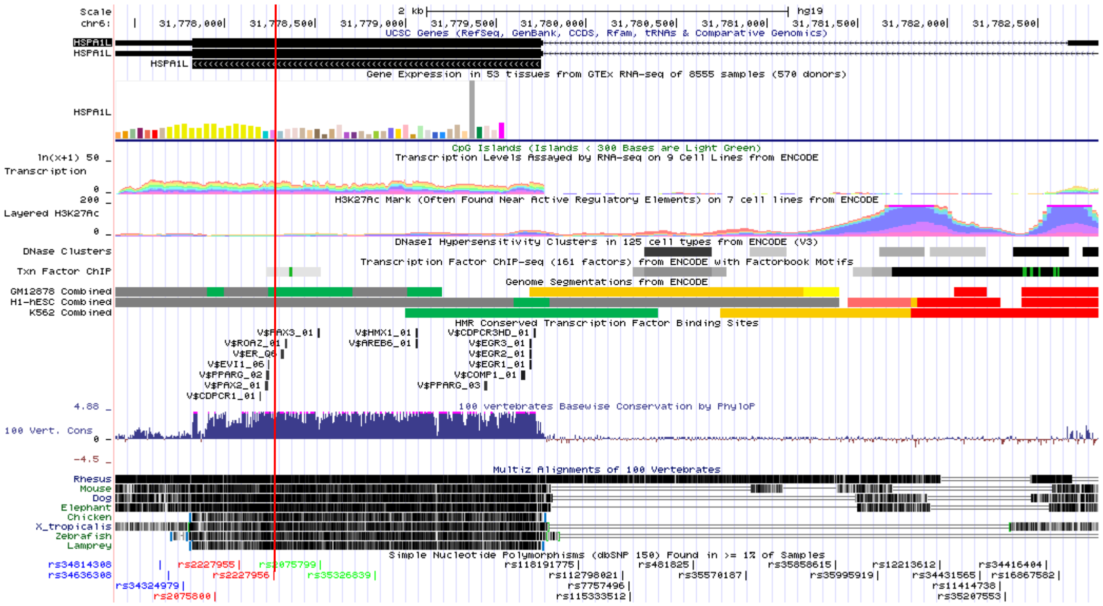

Figure S6 The genomic region of rs2227956 in *HSPA1L* gene

The missense SNP rs2227956 locates in exon 1 of *HSPA1L*. It locates in transcription factor binding sites.

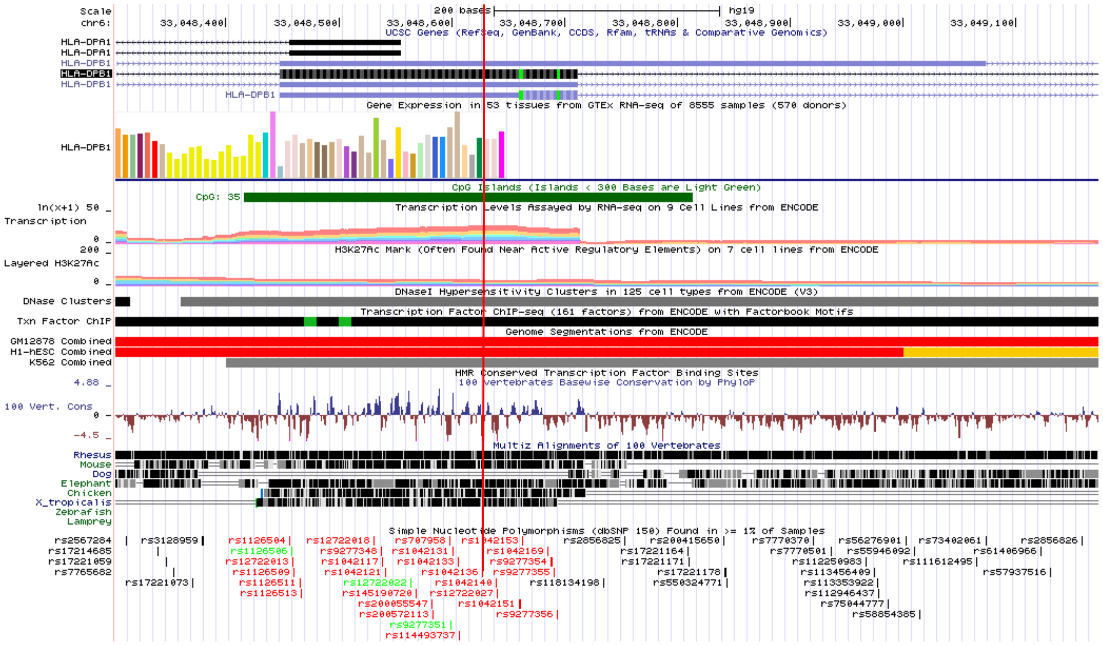

Figure S7 The genomic region of rs1042136 in *HLA-DPB1* gene

The missense SNP rs1042136 locates in exon 2 of *HLA-DPB1*. It locates in a DNase I hypersensitive site and a CpG island.

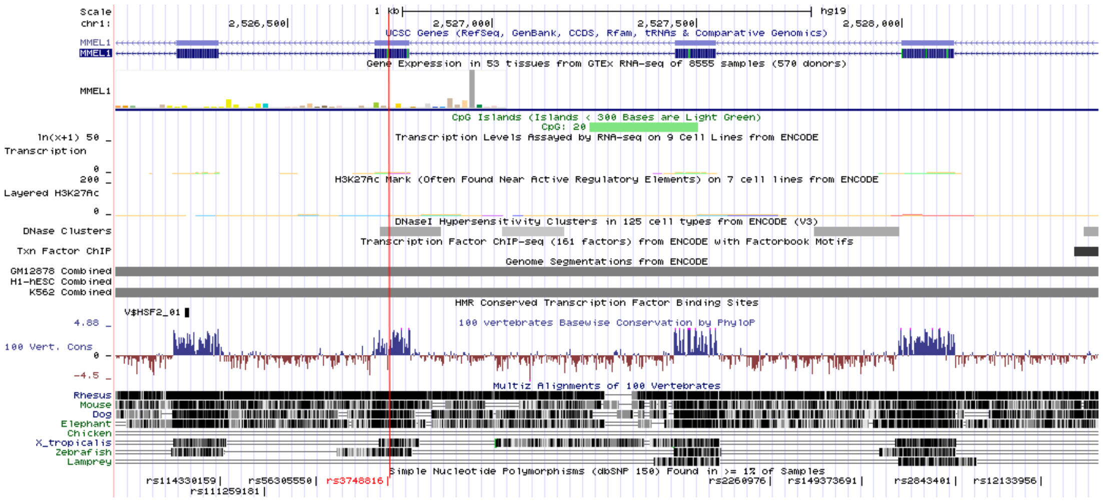

Figure S8 The genomic region of rs3748816 in *MMEL1* gene

SNP rs3748816 locates in an exon of *MMEL1*. This region shows very high conservation, transcription level and DNaseI hypersensitivity. The tracks successively show base position, UCSC gene, gene expression in 53 tissues from GTEx, CpG island, transcription levels assayed by RNA-seq on 9 cell lines from ENCODE, H3K27Ac mark, DNaseI hypersensitivity clusters, transcription factor ChIP-seq from ENCODE, genome segments from ENCODE, HMR conserved transcription factor binding sites, conservation and common SNPs.
